# Probing the pan-genome of *Listeria monocytogenes*: new insights into intraspecific niche expansion and genomic diversification

**DOI:** 10.1186/1471-2164-11-500

**Published:** 2010-09-16

**Authors:** Xiangyu Deng, Adam M Phillippy, Zengxin Li, Steven L Salzberg, Wei Zhang

**Affiliations:** 1 National Center for Food Safety and Technology, Illinois Institute of Technology, Summit, Illinois 60501, USA; 2 Center for Bioinformatics and Computational Biology, University of Maryland, College Park, Maryland 20742, USA

## Abstract

**Background:**

Bacterial pathogens often show significant intraspecific variations in ecological fitness, host preference and pathogenic potential to cause infectious disease. The species of *Listeria monocytogenes*, a facultative intracellular pathogen and the causative agent of human listeriosis, consists of at least three distinct genetic lineages. Two of these lineages predominantly cause human sporadic and epidemic infections, whereas the third lineage has never been implicated in human disease outbreaks despite its overall conservation of many known virulence factors.

**Results:**

Here we compare the genomes of 26 *L. monocytogenes *strains representing the three lineages based on both *in silico *comparative genomic analysis and high-density, pan-genomic DNA array hybridizations. We uncover 86 genes and 8 small regulatory RNAs that likely make *L. monocytogenes *lineages differ in carbohydrate utilization and stress resistance during their residence in natural habitats and passage through the host gastrointestinal tract. We also identify 2,330 to 2,456 core genes that define this species along with an open pan-genome pool that contains more than 4,052 genes. Phylogenomic reconstructions based on 3,560 homologous groups allowed robust estimation of phylogenetic relatedness among *L. monocytogenes *strains.

**Conclusions:**

Our pan-genome approach enables accurate co-analysis of DNA sequence and hybridization array data for both core gene estimation and phylogenomics. Application of our method to the pan-genome of *L. monocytogenes *sheds new insights into the intraspecific niche expansion and evolution of this important foodborne pathogen.

## Background

*Listeria monocytogenes *is a Gram-positive foodborne bacterial pathogen and the causative agent of the human and animal infectious disease, listeriosis. *L. monocytogenes *can thrive in diverse environmental reservoirs (e.g. soil, water, and sewage) and proliferate under unfavorable conditions (e.g. high osmolarity, low pH, and refrigeration temperature) that other bacterial pathogens cannot endure [1-4]. Its robust physiological characteristics, coupled with its ubiquity in food processing, distribution and retail environments, have made *L. monocytogenes *difficult to manage in food manufacturing, particularly for ready-to-eat food products. *L. monocytogenes *causes the highest rates of hospitalization (about 92%) and mortality (about 20%) among all foodborne bacterial pathogens in the United States [5], making the control of this bacterium in foods a high priority for both food safety and public health. Yet, the versatile lifestyle of *L. monocytogenes *both inside and outside its host, and its unique capability to invade and replicate in different host cell types (e.g. macrophages and nonprofessional phagocytes), have made this opportunistic pathogen a paradigm for studying host-pathogen interactions, pathophysiology, gene regulation, and stress adaptation [6,7].

Previous molecular subtyping studies have collectively suggested that the species of *L. monocytogenes *is composed of at least three major evolutionary or genetic lineages that notably differ in their prevalence in causing human and animal diseases [8-15]. Specifically, lineage I (or LI) and lineage II (or LII) of *L. monocytogenes *are frequently isolated from foods and implicated in the vast majority (> 95%) of both sporadic cases and epidemic outbreaks of human listeriosis [3]. Genetic lineage III (or LIII) strains are rarely reported in cases of human infections, but are sometimes associated with animal disease cases [3,14,16]. The mechanisms underlying the biased predominance of certain *L. monocytogenes *genetic lineages in human listeriosis remain largely unknown. Several recent studies have revealed elevated levels of genetic diversity among LIII isolates [12,15]. Multilocus sequence typing analysis on the basis of partial *sigB *and *actA *gene sequences have also suggested that LIII is polyphyletic, with the co-existence of at least three distinct subgroups (i.e. LIIIA, LIIIB, LIIIC) [14,16]. Atypical phenotypes of LIII isolates, such as deficiency in rhamnose fermentation [14], attenuated virulence potential [16], reduced resistance to heat and cold stresses [17] and lowered biofilm productivity [18], have collectively indicated that LIII may have followed a distinct evolutionary path from other *L. monocytogenes *lineages.

Compared to fairly extensive studies on LI and LII strains, little is known about LIII. Although it is documented that most listerial virulence factors such as the positive regulatory factor (or PrfA) are well conserved across the entire *L. monocytogenes *species, LIII strains are underrepresented in both food contamination and human listeriosis. This led us to speculate the existence of additional, yet-to-be-identified genetic factors in the predominant disease-causing *L. monocytogenes *lineages (i.e. LI and LII) that may mediate listerial niche adaptation, resistance to extra- or intracellular stresses, and pathogenicity. These unknown genetic factors may have been lost, mutated, or decayed in LIII as the genomes evolved, resulting in a defective phenotype for LIII isolates in certain ecological and host niches. To test our hypothesis, we combined *in silico *comparative genomic analyses with an array-based comparative genomic hybridization (CGH) approach to probe the genomic diversity of *L. monocytogenes *and to identify genomic features common in LI and LII but absent in LIII. Array CGH is a powerful yet cost-effective approach for genotyping and detecting intraspecies genomic diversity for many bacteria. Previous efforts on comparative genomic analyses underscore the usefulness of CGH in resolving genetic lineages and identifying strain- or lineage-specific genes in *L. monocytogenes *[10,19-22]. However, most of these studies targeted only a number of selected genes or partial listerial genomes, making an accurate assessment of intraspecies genomic diversity difficult.

It is recognized that a few sequenced genomes may not fully represent the entire genetic repertoire of a given organism [23-35]. For this reason, the pan-genome concept has triggered new investigations on genomic diversity for several bacterial species, including *Streptococcus spp. *[24,28,29], *Haemophilus influenzae *[25], *Neisseria meningitides *[30], *Escherichia coli *[31-33], and *Lactococcus lactis *[34]. Pan-genome refers to the total genetic repertoire of a given species, which is typically composed of "core" genes plus some "dispensable" or "accessory" genes [25,27,35]. Pan-genomic DNA arrays, which probe the full genetic repertoire, have recently gained increasing popularity for the systematic survey of diversity in prokaryotic species [31,36,37].

The availability of more than 20 *L. monocytogenes *full and draft genomes has made this pathogen an ideal candidate for pan-genomic study (Table 1). Our initial comparative analysis of 17 *L. monocytogenes *genomes indicated a "closed" pan-genome for this bacterial species. Species with a closed pan-genome typically share highly syntenic genomes with less frequent horizontal gene transfers (HGT) and genomic rearrangements. Therefore, the entire gene pool can be fully sampled by sequencing a small set of representative isolates, and the number of new genes to be discovered by sequencing additional genomes will quickly approach zero. This prompted us to design and construct a pan-genome CGH array that, in theory, accommodates the total genomic diversity of the *L. monocytogenes *species on a single DNA chip. Compared to several previous pan-genome microarrays that targeted either the conserved sequence of gene families with low probe density or no coverage of the intergenic regions, we utilized a novel probe selection algorithm (PanArray) to design a pan-genome tiling array that incorporates the genomes of 20 available *L. monocytogenes *strains [38]. This design provides unbiased coverage of the pan-genome, and also superior accuracy and resolution for data analysis.

**Table 1:** *L. monocytogenes *genomes analyzed in this study

Strain	Lineage	Serotype	Size (bp)	**Contigs**^**1**^	**Genes**^**2**^	**% Identity**^**3**^	Genbank Accession	Sequencing institution	**Note**^**4**^
EGD-e	II	1/2a	2,944,528	Closed	2931	100	AL591824	European consortium [43]	DSA
R2-561	II	1/2c	2,945,851	37	2993	99.78	AARS00000000	Broad Institute	DS
LO28	II	1/2c	2,675,580	1150	3030	99.6	AARY00000000	Broad Institute/Institut Pasteur	D
Finland 1988	II	3a	2,834,040	49	2740	98.49	AART00000000	Broad Institute	S
10403S	II	1/2a	2,873,541	21	2905	98.48	AARZ00000000	Broad Institute	DS
F2-515	II	1/2a	1,815,995	1728	2710	98.47	AARI00000000	Broad Institute	D
N3-165	II	1/2a	2,884,080	39	2885	98.39	AARQ00000000	Broad Institute	DS
J2-003	II	1/2a	2,741,640	795	2972	98.32	AARM00000000	Broad Institute	D
F6900	II	1/2a	2,968,620	23	3007	98.28	AARU00000000	Broad Institute	DS
F6854	II	1/2a	2,950,285	133	2967	98.26	AADQ00000000	TIGR	DS
J2818	II	1/2a	2,973,040	24	3020	98.24	AARX00000000	Broad Institute	DS
J0161	II	1/2a	3,062,582	25	3114	98.23	AARW00000000	Broad Institute	DS
J1-175	I	1/2b	2,866,484	457	3178	94.39	AARK00000000	Broad Institute	D
J2-064	I	1/2b	2,828,700	545	2968	94.37	AARO00000000	Broad Institute	D
R2-503	I	1/2b	2,991,493	55	2968	94.28	AARR00000000	Broad Institute	S
J1-194	I	1/2b	2,989,818	30	3040	94.27	AARJ00000000	Broad Institute	DS
N1-017	I	4b	3,142,060	79	3253	94.2	AARP00000000	Broad Institute	DS^5^
Clip 80459	I	4b	2,912,690	Closed	2972	94.17	FM242711	Institut Pasteur	S
F2365	I	4b	2,905,187	Closed	2907	94.14	AE017262	TIGR [85]	DS
H7858	I	4b	2,972,254	181	3195	94.08	AADR00000000	TIGR	DS
HPB2262	I	4b	2,991,120	79	3067	93.98	AATL00000000	Broad Institute/Istituto Superiore di Sanita	DS
HCC23	III	4a	2,976,212	Closed	3059	92.38	CP001175	Mississippi State University	S
F2-524	IIIA	4a	-	-	-	-	-	-	A
F2-501	IIIA	4b	-	-	-	-	-	-	A
J2-071	IIIA	4c	2,851,800	53	2778	92.6	AARN00000000	Broad Institute	DA^5^
J1-208	IIIB	4a	1,963,740	1660	2809	91.8	AARL00000000	Broad Institute	DA
M1-002	IIIB	4b	-	-	-	-	-	-	A
W1-111	IIIB	4c	-	-	-	-	-	-	A
F2-208	IIIC	4a	-	-	-	-	-	Life Technologies Corporation/Cornell University	A
F2-569	IIIC	4b	-	-	-	-	-	-	A
W1-110	IIIC	4c	-	-	-	-	-	-	A

^1^Number of contigs based on GenBank at the time of our study. Strains with > 200 contigs were sequenced only to low coverage and were excluded from analysis.

^2^Number of annotated protein coding genes and RNAs based on GenBank.

^3^Nucleotide sequence identity in reference to EGD-e.

^4^Strains used for array design (D); comparative sequence analysis (S), comparative genomic hybridizations (A).

^5^Strains N1-017 and J2-071 were found to be mislabeled in GenBank; this has since been fixed.

- Information not available.

Using integrated data obtained from both *in silico *whole-genome comparisons and pan-genome CGH analyses, we (*1*) explored the intraspecific genetic diversity of *L. monocytogenes *with a focus on the largely unexplored genetic lineage III; (*2*) estimated the core and pan-genome that define the *L. monocytogenes *species; (*3*) identified unique protein-coding genes and regulatory RNAs in the predominant disease-causing lineages, as they may relate to ecological fitness, host niche adaptation and pathogenicity; and (*4*) reconstructed phylogeny for different *L. monocytogenes *lineages and strains based on pan-genome characteristics.

## Results

### Pan-genomic array coverage

Initial power-law regression analysis of 17 sequenced *L. monocytogenes *genomes (Table 1) suggested that this bacterial species exhibits a nearly closed pan-genome, which would yield rapidly diminishing returns of less than 7 novel genes per additional genome sequenced. Therefore, we presumed a single array could be designed to query the full genetic repertoire of the species, and be used to completely genotype currently unsequenced strains. For this purpose we designed a pan-genomic array comprising 385,000 50-mer *in situ *synthesized oligonucleotide probes that fully tile the sequences of 20 *L. monocytogenes *genomes (Table 1), with no gaps, at greater than 2-fold coverage of each genome. Shortly after we completed our chip design, four additional *L. monocytogenes *genomes were sequenced to closure, including strain Clip 80459 (LI), strain Finland 1988 (LII), strain R2-561 (LII) and strain HCC23 (LIII). These new *L. monocytogenes *genomes enabled us to evaluate the genomic coverage of our array design by individually mapping each of the 385,000 oligonucleotide probes to annotated genes to the four genomes. A 50-mer probe was mapped to a particular gene if it perfectly matched the gene sequence or contained only a single nucleotide mismatch. For each annotated gene, the probe coverage was calculated as the percentage of the gene length covered by mapped probes. Results in Table 2 suggest that our design adequately represents the intraspecies diversity of *L. monocytogenes*, particularly for LI and LII genomes. However, due to the limited number of fully sequenced LIII genomes available at the time of design, the coverage for LIII specific genes is less optimal, as indicated by HCC23.

**Table 2:** Probe coverage of newly sequenced genomes

Genome	Lineage	Probe coverage		
		100%	90%	80%
R2-561	II	0.95	0.98	0.98
Clip 80459	I	0.91	0.99	0.99
Finland 1988	I	0.80	0.96	0.98
HCC23	III	0.30	0.80	0.89

Proportion of genes from four newly sequenced strains with probe coverage meeting a minimum percentage of the gene length (100%, 90%, 80%) for probes containing at most one SNP.

### Accuracy of CGH detection calls

**Figure 1:**
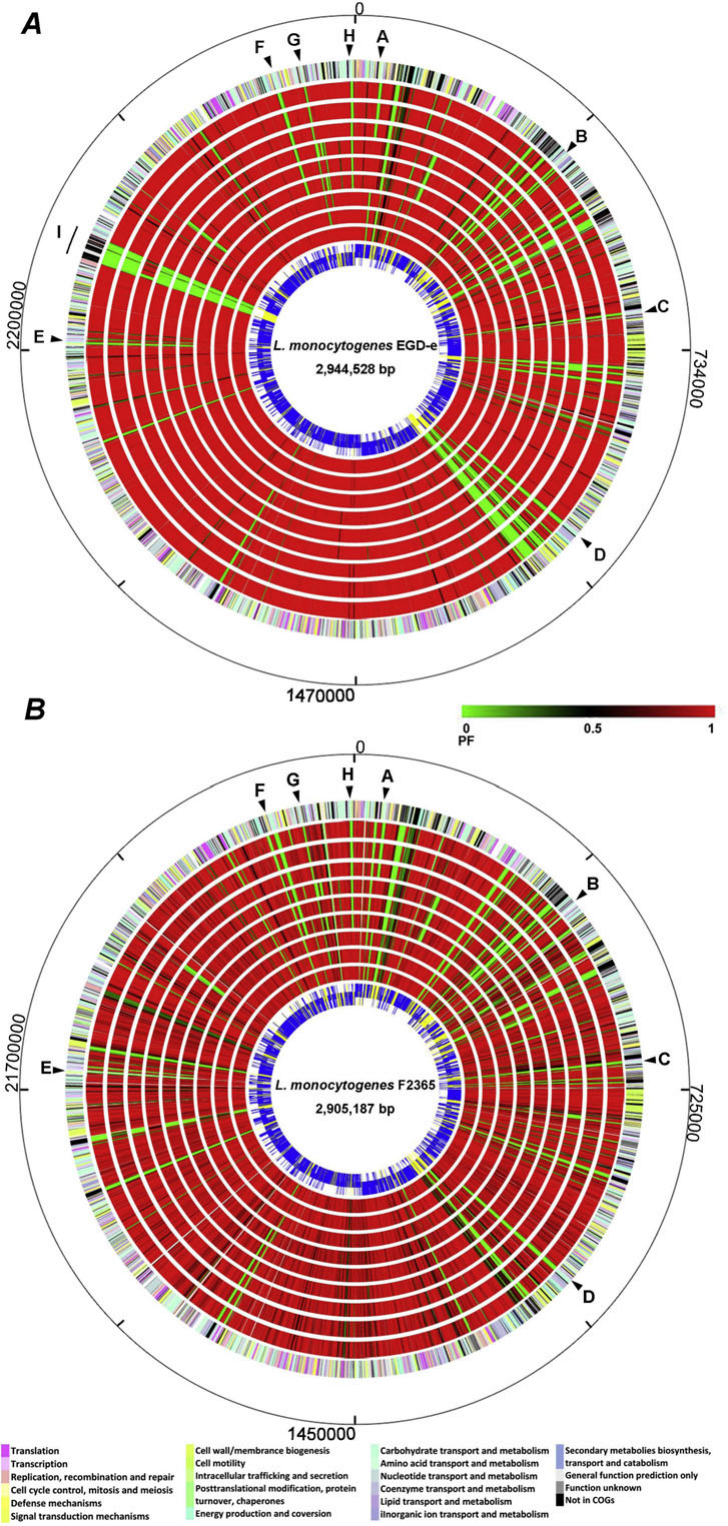
**Circular maps that compare the genomes of nine *L. monocytogenes *LIII strains with that of a LII reference strain EGD-e (*A*) and a LI reference strain F2365 (*B*)**. The inner most circle is the reference genome. Core genes in the reference genome are shown blue and accessory genes are shown in yellow. From inside out, the second to the tenth circles represent the nine LIII genomes, including J2-071 (LIIIA), F2-501 (LIIIA), F2-504 (LIIIA), J1-208 (LIIIB), M1-002 (LIIIB), W1-111 (LIIIB), F2-208 (LIIIC), F2-569 (LIIIC), and W1-110 (LIIIC), respectively. Genes in LIII genomes are color-coded based on the PF values (see the reference bar). Green indicates a gene is absent (PF = 0) in a LIII genome; red indicates a gene is conserved (PF = 1) in a LIII genome at the corresponding location in the reference genome. The eleventh circle gives color-coded gene annotations in the reference genome based Clusters of Orthologous Groups of proteins (see the color codes at the bottom). The outer most circle provides relative genomic coordinates. Eight DDG clusters at similar genomic locations in EGD-e and F2365 are marked with letters A through H. Specifically: A, *lmo0037-0041 *(or *lmof2365_0045-0050*); B, *lmo0357-0360 *(or *lmof2365_0377-0381*); C, *lmo0631-0633 *(or *lmof2365_0660-0662*); D, *lmo1030-1036 *(or *lmof2365_1051-1057*); E, *lmo2133-2138*; F, *lmo2732-2736 *(or *lmof2365_2719-2723*); G, *lmo2771-2773 *(or *lmof2365_2761-2763*); and H, *lmo2846-2851 *(or *lmof2365_2836-2841*), respectively. The LII-specific *comK *prophage integration region was marked in the EGD-e genome (I). The figure was created using GenomeViz.

Genomic DNA of nine LIII strains were each co-hybridized on the pan-genome arrays with that of EGD-e (LII) as an internal reference. The nine LIII strains were carefully selected from a strain collection to represent 3 different serotypes (4a, 4b, and 4c) and 3 different subgroups (LIIIA, LIIIB, and LIIIC) of this lineage (Table 1). Individual probes were designated as present or absent in the sample based on statistical analysis of the normalized signal intensities (see Materials and Methods). Since the position of each probe is known for all sequenced *L. monocytogenes *genomes, genes were scored by the fraction of targeting probes with a positive signal, otherwise known as the positive fraction (PF). This yields a very flexible scoring scheme that can be readily applied to any intragenic or intergenic feature of the genome targeted by a sufficient number of probes. A high PF indicates a gene is likely present in the hybridized genome. Circular maps of all PF values for the nine LIII genomes in reference to an LI strain F2365 and an LII strain EGD-e are shown in Figure 1.

**Figure 2:**
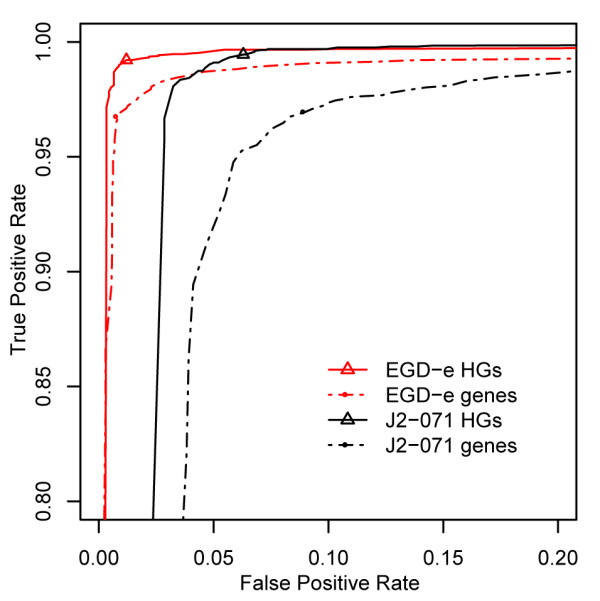
**Receiver operating characteristic curves**. ROC curves compare true-positive rates with false-positive rates of different PF cutoffs for prediction of the presence or absence of individual gene variants and homologous groups. Error rates are shown for genes (dotted lines) and homologous groups (solid lines), computed from EGD-e (red) and J2-071 (black) control hybridizations. Circles indicate the chosen PF cutoff of 0.6 for classifying gene variants. Triangles indicate the chosen PF cutoff of 0.6 for classifying homologous groups.

To select an appropriate PF threshold and test the accuracy of gene calls based on PF values, we examined the true-positive and false-positive rates of the PF criterion for 51,814 annotated *L. monocytogenes *genes, compared against genomes for which we had both sequence and CGH array data. True gene "presence" was determined by a tblastn search of the 51,814 predicted proteins against a six frame translation of the genome [39], requiring a minimum of 50% amino acid similarity and an *E*-value ≤ 10^-5^. Figure 2 shows the Receiver Operating Characteristics (ROC) curves for the PF criterion measured against the tblastn standard for two *L. monocytogenes *strains, EGD-e and J2-071. The PF measure is remarkably robust, as there appear to be very few genes near the classification threshold. Additional file 1 shows a density estimation of PF values for both present and absent genes, suggesting that the vast majority of present genes have PF > 0.9 and absent genes PF < 0.1. Based on the ROC analysis, a PF cutoff of 0.6 was chosen to best match the tblastn results and minimize the expected error rate. The seemingly higher false-positive rate for J2-071, in comparison to the closed EGD-e genome, is partially due to tblastn false-negatives incurred from the 78 gaps in the J2-071 draft genome. In these cases, a gene that is truly present, but overlapping a sequencing gap, is falsely reported as absent by the tblastn method which artificially increases the measured false-positive rate of the CGH array method.

Accuracy statistics for the chosen 0.6 PF cutoff versus the 50% alignment similarity cutoff are given in Table 3. The array has perfect sensitivity for detecting the EGD-e and J2-071 control genes. Expected accuracy was estimated for detecting both individual gene variants from all other strains and for detecting homologous gene groups (HGs). Orthologous gene groups are typically preferred; however, the inability of CGH to accurately determine sequence identity and gene order makes it impractical to discriminate between highly similar paralogs. Alternatively, we tested for the presence of 3,560 strongly homologous groups, identified by clustering proteins with higher than 50% amino acid similarity. A gene group was marked as present in a genome if any gene from that group exceeded the BLAST or PF threshold. Figure 2 also displays the true- and false-positive rates of HG detection alongside the original ROC curves. In comparison to detecting individual gene variants, HG detection significantly increases the sensitivity of the array without increasing the false-positive rate. When analyzing only a single gene variant on the chip, high polymorphism in the sample genome can disrupt hybridization and lead to false-negatives. However, by considering an entire gene group, a sample only needs to hybridize with its nearest variant, thereby increasing the sensitivity [34]. To demonstrate the sensitivity of the array at detecting HGs in unsequenced strains, Table 3 also lists accuracy statistics for EGD-e and J2-071 when the probes specific to those genomes are removed from the analysis. This simulates the accuracy of the array at calling genes in an unsequenced LII or LIII strain. The sensitivity of the array is only slightly affected, with a 0.2% true-positive rate drop for EGD-e and a 1.3% drop for J2-071. The drop is more pronounced for J2-071 because it is one of only two LIII genomes included on the array, so ignoring the J2-071 specific probes affects the sensitivity of calling HGs from that lineage.

**Table 3:** Accuracy of the pan-genome array for detecting genes and homologous groups

Chip Data	Test Data	Present	Absent	ACC	TPR	FPR	FDR
EGD-e	EGD-e genes only	2846	0	1.000 ± 0.000	1.000 ± 0.000	N/A	N/A
EGD-e	All gene variants	49068	2746	0.973 ± 0.002	0.973 ± 0.003	0.020 ± 0.009	0.001 ± 0.000
EGD-e	Gene groups	2642	918	0.989 ± 0.002	0.993 ± 0.001	0.024 ± 0.007	0.008 ± 0.003
EGD-e(-)	Gene groups	2627	918	0.987 ± 0.002	0.991 ± 0.001	0.024 ± 0.007	0.008 ± 0.003
J2-071	J2-071 genes only	2694	0	1.000	1.000	N/A	N/A
J2-071	All gene variants	47411	4403	0.964	0.970	0.090	0.009
J2-071	Gene groups	2543	1017	0.978	0.995	0.063	0.025
J2-071(-)	Gene groups	2468	1016	0.969	0.982	0.062	0.025

Present/Absent are based on a tblastn search. ACC, TPR, FPR, FDR stand for accuracy, true-positive rate, false-positive rate, and false discovery rate, respectively. (-) Excludes all probes directly targeting the hybridized strain from the analysis to simulate detection accuracy for an unknown strain. For EGD-e, the mean of 9 data sets are given, along with their standard deviation to illustrate array reproducibility.

### Estimation of core and pan-genomes

The expected number of new genes to be discovered by sequencing additional *L. monocytogenes *strains, and the sizes of the core and pan-genomes, were estimated using methods adapted from Tettelin *et al. *[24]. Frequent gaps and sequencing errors in low-quality genome assemblies were found to cause many missed protein alignments, which affected the core genome estimation. For example, only 683 EGD-e proteins meet the alignment threshold in all 24 draft *L. monocytogenes *genomes, an unreasonably low number. Additionally, fragmented annotations in the low quality genomes artificially inflate the pan-genome size estimate. To avoid these artifacts, only 18 "high quality" *L. monocytogenes *genomes were used for the new genes and pan-genome estimation. Genomes sequenced to less than 10× coverage using 454 pyrosequencing were excluded from the sequence analysis (Table 1). Array CGH results for the 8 additional LIII genomes were included in the core gene estimate.

**Figure 3:**
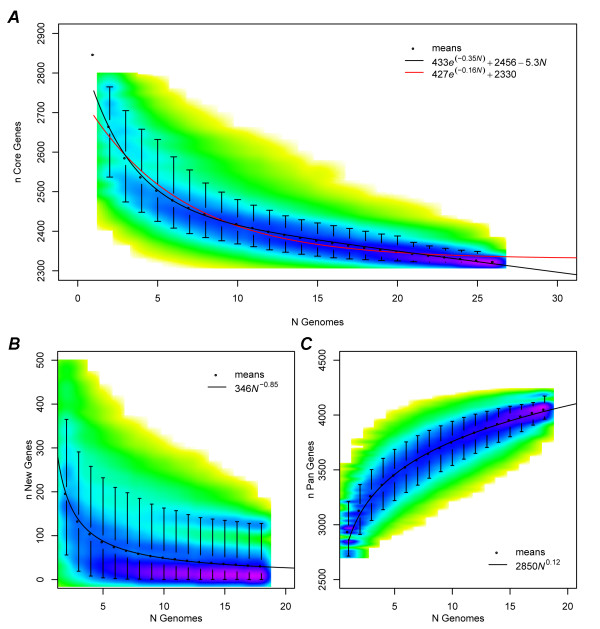
**Prediction of core, new and pan genes in *L. monocytogenes***. (*A*) Exponential regression analysis that predicts the number of core genes in *N *sequenced genomes. For each *N*, permutations are randomly sampled and the number of core genes conserved in all *N *genomes is computed. The estimated number of core genes in 26 *L. monocytogenes *genomes ranges from 2,330 to 2,456. The sampled distribution is represented by a smoothed color density plot obtained through kernel density estimation. Yellow indicates the lowest density and purple indicates the highest density. For each *N*, black circles indicate the mean value and whiskers indicate the 5^th ^and the 95^th ^percentiles of the distribution. An exponential decay fit to the means is given by a solid red curve. A modified exponential decay is given by a solid black curve, which better fits the observed data by accounting for false-negative gene calls. (*B*) Power law regression analysis predicts the number of new genes that will be discovered by sequencing additional *L. monocytogenes *genomes. The LIII genomes are the outliers that pull the means higher, indicating that LIII diversity has not yet been fully sequenced. (*C*) Power law regression analysis predicts the number of *L. monocytogenes *pan genes accumulated from genome sequencing is currently 4,052 and growing.

To estimate the *L. monocytogenes *core genome, the number of shared genes was computed for many random permutations of *N *genomes, and the mean number of shared genes was computed for each *N*. The number of core genes for the species was estimated by fitting an exponential decay function to the means. For the high-quality sequenced genomes, this analysis yielded an estimated horizontal asymptote of 2,467 ± 7 core genes. However, the sequenced genomes include only two LIII genomes. Repeating the analysis for all 26 genomes, including CGH results for the 8 additional LIII genomes, reduced the estimate by over 100 genes to 2,330 ± 5, emphasizing the importance of a balanced sample of diversity for estimating core genome size. Figure 3A displays the result of the 26 genome analysis including a smoothed density plot of the shared gene count distributions, the mean value for each *N*, and the best-fit exponential decay.

Imperfect detection sensitivity due to sequencing gaps makes it impossible to achieve convergence for real data, so an exact core genome cannot be determined. Any non-zero false-positive rate for detecting core genes will artificially shrink the core genome with each additional genome, violating the horizontal asymptote of an exponential decay. This is evident in the almost linearly decreasing means towards the tail of Figure 3A. To account for these false-negatives, we introduced an additional parameter to the core genes model that adds a constant number of false-negatives upon the addition of each genome (see Materials and Methods). The revised model is a much closer fit to the data (residual standard error of 2.98 versus 10.68), accounts for noisy draft and CGH data, and yields an increased core genes estimate of 2,456 ± 4. This likely represents an upper bound on the core genome size. Considering results from both models, and the uncertainty caused by the draft genomes and CGH data, we estimate the core genome of *L. monocytogenes *to be between 2,330 to 2,456 genes (approximately 80% of a typical *L. monocytogenes *genome).

A major limitation of array CGH is that this method cannot detect novel genes contained in LIII genomes. For this reason, the pan-genome estimation was performed for only the high-quality sequenced genomes, of which two are from LIII. Again, the number of new genes identified by sequencing each additional genome was computed for many random permutations of *N *genomes. The number of new genes identified for each *N *was modeled by the power law function *n *= *κN*^-*α *^[26]. Using the *median *values, the power law exponent *α *was estimated to be 1.12 ± 0.02. This is slightly lower than our original estimate of 1.38 due to the recent sequencing of four additional genomes, an updated annotation, and a stricter similarity threshold. In both cases, an exponent *α *> 1 indicates a closed pan-genome, meaning the size of the pan-genome is a bounded function of the number of sequenced genomes. However, fitting a power law to the *mean *values of these distributions yields *α *= 0.85 ± 0.01, suggesting an open pan-genome (Figure 3C). This difference is caused largely by the diverse strains N1-017, HCC23, and J2-071, which contain many strain-specific genes and pull the mean values higher than the medians. For example, strain HCC23 contains 122 strain-specific genes not found in any of the other 17 strains. Removal of these three genomes from the analysis results in an *α *slightly greater than one for both the mean and median analyses. Two of these genomes are the only LIII strains in the analysis, indicating that additional sequencing from LIII may reduce the exponent even further. This regression analysis suggests *L. monocytogenes *has a significantly diverse gene reservoir, and additional sequencing of LIII genomes is necessary to resolve the exact size and nature of the *L. monocytogenes *pan-genome.

The estimated growth of the *L. monocytogenes *pan-genome with additional sequencing was also simulated using many random permutations of genomes. For open pan-genomes, the cumulative number of unique genes discovered with the sequencing of additional genomes can be modeled by Heap's law using the power law function *n *= *κN*^*γ *^[26]. This regression is illustrated by Figure 3B and *γ *was estimated as 0.12 ± 0.001. Since the growth of an open pan-genome is equivalent to the number of new genes added after sequencing each successive genome, the derivative of the pan genes function should be equal to the new genes function. That is *N*^*γ*-1 ^∝ *N*^-*α *^and *α *= 1 - *γ *for *α *< 1. Although simulated separately, the pan and new gene functions do follow this property for the mean value regressions, with *α *= 0.85 and *γ *= 0.12 being in good agreement. For *N *= 18, the mean estimated pan-genome size is 4,052 and continues to grow, with diminishing returns, for larger *N*.

The above method is useful for estimating the size of the pan-genome, but because it depends on the order of the genomes analyzed, it does not yield a single representative set of pan genes for the analyzed strains. An alternative that does not depend on the order of genomes is to measure the number of gene groups identified by a similarity clustering method such as OrthoMCL [40]. We applied a similar approach, but for clustering strong homologs rather than orthologs, to be consistent with the other analyses. From a graph of 52,776 proteins with > 50% similar proteins connected by edges, 3,744 HGs were identified (Additional file 2) using the MCL graph clustering algorithm [41]. This provides a relative lower bound for the size of the currently sequenced *L. monocytogenes *pan-genome.

### Lineage-specific genes and disparately distributed genes

Lineage-specific genes refer to genes that are exclusively present in a single *L. monocytogenes *lineage based on the above defined similarity threshold. To maintain a stringent threshold, a gene is not considered to be lineage-specific if any member of its HG is present in another lineage. Annotated genes in F2365 (LI), EGD-e (LII), and J2-071 (LIII) were used to screen for gene lineage specificity against all genomes analyzed in this study. Table 4 lists 4 LI- 5 LII- and 6 LIII-specific genes identified in our study. Most of these genes encode hypothetical proteins. It is notable that only 5 of the 21 LII-specific genes previously identified by Doumith *et al *[10] passed our lineage specificity threshold. We used colony polymerase chain reaction (PCR) assays to verify the lineage specificity for all LI- and LIII-specific genes identified by CGH analysis (except for LMOf2365-0409 due to the small size of this gene for proper PCR primer design). A total of 225 colony PCR assays were conducted for randomly selected *L. monocytogenes *strains in our collection, including 8 LI, 8 LII, and 9 LIII strains. The PCR results confirmed the lineage specificity for all genes analyzed, suggesting that the CGH approach was accurate for calling gene presence or absence and determining lineage specificity.

**Table 4:** Lineage specific genes in *L. monocytogenes*

Gene	Genome	Annotation
**Lineage I specific**		
*LMOf2365_0409*	F2365	Hypothetical protein
*LMOf2365_1251*	F2365	Hypothetical protein
*LMOf2365_1252*	F2365	Hypothetical protein
*LMOf2365_2638*	F2365	Similar to cell surface anchor family protein
**Lineage II specific**		
*lmo0525*	EGD-e	Hypothetical protein
*lmo0737*	EGD-e	Hypothetical protein
*lmo1061*	EGD-e	Similar to two-component sensor histidine kinase
*lmo1968*	EGD-e	Similar to creatinine amidohydrolases
*lmo1969*	EGD-e	Similar to 2-keto-3-deoxygluconate-6-phosphate aldolase
**Lineage III specific**		
*LmonocytogFSL_030100003416*	J2-071	Hypothetical protein
*LmonocytogFSL_030100004481*	J2-071	Hypothetical protein
*LmonocytogFSL_030100010091*	J2-071	Similar to ADP-ribose 1''-phosphate domain protein
*LmonocytogFSL_030100010130*	J2-071	Hypothetical protein
*LmonocytogFSL_030100011357*	J2-071	Hypothetical protein
*LmonocytogFSL_030100012027*	J2-071	Hypothetical protein

Lineage specificity is based on comparative analysis of 26 genomes in this study, including 7 LI strains (F2365, H7858, Clip 80459, N1-017, R2-503, HPB2262 and J1-194), 9 LII strains (EGD-e, R2-561, Finland 1988, 10403S, N3-165, F6900, F6854, J2818 and J0161) and 10 LIII genomes (HCC23, J2-071, F2-501, F2-524, J1-208, M1-002, W1-111, F2-208, F2-569 and W1-110). Gene ID is designated based on a respective reference genome.

We identified 86 disparately distributed genes (or DDGs) as listed in Table 5. DDGs refer to genes that are highly conserved (PF > 0.6 or protein similarity >50%) in LI and LII genomes but absent or highly divergent (PF < 0.6) in at least six of the nine LIII genomes. DDGs are of particular interest for us because the biased distribution and conservation of these genes in LI and LII genomes likely correlate to the enhanced ecological fitness and pathogenicity of *L. monocytogenes *in the host. The largest functional group of DDGs (41%) is associated with carbohydrate transport and metabolism. Figure 1 illustrates their distribution. *L. monocytogenes *harbors one of the largest bacterial carbohydrate phosphotransferase system (PTS) genes [42-44]. The abundance and diversity of the PTS system allows this soil saprophyte to utilize different carbon sources associated with the ecosystems it inhabits such as soil, silage and sediments. Fifteen PTS genes were identified as DDGs; most are associated with fructose-specific PTS enzyme II components (*lmo0357-0358*, *lmo0631-0633*, *lmo2135-2137*, and *lmo2733*). We surveyed the distribution of 978 annotated PTS genes and their homologs in all 26 *L. monocytogenes *genomes, and found 965 (99%) PTS genes are conserved in all LI and LII genomes and 7 (0.7%) are specific to LI. In contrast, 137 (14%) PTS genes are absent or divergent in LIII genomes. Diversity in PTS content is most noticeable among the three LIII subgroups, where 48 (4.8%), 137 (14%), and 136 (13.9%) PTS genes are absent in LIIIA, LIIIB and LIIIC, respectively. An interesting distinction among 3 subgroups is that LIIIA strains are capable of fermenting rhamnose, whereas LIIIB and LIIIC strains are deficient in rhamnose utilization [14]. We discovered a cluster of six genes (*lmo2846-2851*), which is likely to mediate rhamnose utilization, is missing from all LIIIB and LIIIC genomes. Five genes in this cluster [45] share protein similarities to the rhamnose catabolic pathway in *Escherichia coli *[46,47] and other Gram-positive bacteria such as *Bacillus subtilius *(Additional file 3).

**Table 5:** Genes that are conserved in LI and LII but absent or disparately distributed in LIII

**Gene**^**1**^	Annotation	**LIII**^**2**^	***L. innocua***^**3**^	**Operon**^**4**^
**Carbohydrate transport and metabolism**				
*lmo0357*	Similar to PTS system, enzyme IIA component	IIIA	+	059
*lmo0358*	Similar to PTS system, fructose-specific enzyme IIBC component	IIIA	+	059
*lmo0359*	Similar to D-fructose-1,6-biphosphate aldolase"	IIIA	+	-
*lmo0631*	Similar to PTS system, fructose-specific IIA component	IIIA	-	-
*lmo0632*	Similar to PTS system, fructose-specific IIC component	IIIA	+	-
*lmo0633*	Similar to PTS system, fructose-specific IIB component	IIIA	+	-
*lmo0735*	Similar to ribulose-5-phosphate 3-epimerase	IIIA	+	119
*lmo0736*	Similar to ribose 5-phosphate isomerase	IIIA	+	119
*lmo0738*	Similar to PTS system, beta-glucoside-specific enzyme IIABC	IIIA	+	119
*lmo0739*	Similar to 6-phospho-beta-glucosidase	IIIA	+	119
*lmo0766*	Similar to putative sugar ABC transporter, permease protein	IIIA	+	-
*lmo0767*	Similar to ABC transporter, permease protein	IIIA	+	-
*lmo1031*	Hypothetical protein	IIIA	-	166
*lmo1032*	Similar to transketolase	IIIA	-	166
*lmo1033*	Similar to transketolase	IIIA	-	166
*lmo1035*	Similar to PTS beta-glucoside-specific enzyme IIABC	IIIA	+	166
*lmo1971*	Similar to pentitol PTS system enzyme II C component	IIIA	+	-
*lmo1972*	Similar to pentitol PTS system enzyme II B component	IIIA	+	-
*lmo1973*	Similar to PTS system enzyme II A component	IIIA	+	-
*lmo2133*	Similar to fructose-1,6-biphosphate aldolase type	IIIA	+	-
*lmo2134*	Similar to fructose-1,6-biphosphate aldolase type II	IIIA	+	-
*lmo2135*	Similar to PTS system, fructose-specific enzyme IIC component	IIIA	+	-
*lmo2136*	Similar to PTS system, fructose-specific enzyme IIB component	IIIA	+	-
*lmo2137*	Similar to PTS system, fructose-specific enzyme IIA component	IIIA	+	-
*lmo2143*	Similar to mannose-6-phosphate isomerase	IIIA	-	-
*lmo2733*	Similar to PTS system, fructose-specific IIABC component	IIIA	+	494
*lmo2734*	Similar to sugar hydrolase	IIIA	+	494
*lmo2735*	Similar to Sucrose phosphorylase	IIIA	+	494
*lmo2736*	Hypothetical protein	IIIA	+	494
*lmo2771*	Similar to beta-glucosidase	IIIA	+	-
*lmo2772*	Similar to PTS system, beta-glucoside-specific enzyme IIABC	IIIA	+	-
*lmo2847*	Similar to rhamnulose-1-phosphate aldolase	IIIA	+	516
*lmo2848*	Similar to L-rhamnose isomerase	IIIA	+	516
*lmo2849*	Similar to rhamnulokinase	IIIA	+	516
*lmo2850*	Similar to sugar transport proteins	IIIA	+	516
**Cell envelope biogenesis, outer membrane**				
*lmo0017*	Similar to *Bacillus anthracis *CapA protein	IIIA	-	-
**Cell wall**				
*lmo0933*	Similar to sugar transferase	IIIA	+	-
*lmo1062*	Similar to ABC transporters (permease protein)	IIIA	+	-
*lmo1088*	TagB, teichoic acid biosynthesis protein B precursor	IIIA	+	177
*lmo1089*	TagD, teichoic acid biosynthesis protein D	IIIA	+	177
*lmo0333*	Similar to internalin, putative peptidoglycan bound protein	IIIA	-	-
*lmo0842*	Putative peptidoglycan bound protein (LPXTG motif)	IIIA	+	-
*lmo1136*	Similar to internalin, putative peptidoglycan bound protein	IIIA	+	-
*lmo1289*	Similar to internalin, putative peptidoglycan bound protein	IIIA	+	-
*lmo1666*	Peptidoglycan linked protein (LPXTG motif)	IIIA	-	-
*lmo2085*	Putative peptidoglycan binding protein (LPXTG motif)	IIIA	-	-
*lmo2026*	Putative peptidoglycan binding protein (LPXTG motif)	IIIA	+	-
*lmo2550*	Similar to glycosyl transferases	IIIA	+	-
**Energy production and conversion**				
*lmo0334*	Hypothetical protein	IIIA	-	-
*lmo1034*	Similar to glycerol kinase	IIIA	+	166
**General function prediction only**				
*lmo0752*	Weakly similar to a putative haloacetate dehalogenase	IIIA	-	-
*lmo1970*	Similar to putative phosphotriesterase related proteins	IIIA	+	-
**Phage-related**				
*lmo2285*	Protein gp18, bacteriophage A118	IIIA	+	-
*lmo2286*	Protein gp17, bacteriophage A118	IIIA	+	-
**Secondary metabolites biosynthesis**				
*lmo2157*	SepA, required for septum formation	IIIA	-	-
**Acid and bile resistance**				
*lmo0037*	Similar to amino acid transporter	IIIB	-	-
*lmo0038*	Similar to agmatine deiminase	IIIB	-	008
*lmo0039*	Similar to carbamate kinase	IIIB	-	008
*lmo0040*	Similar to agmatine deiminase	IIIB	-	-
*lmo0447*	Similar to glutamate decarboxylase	IIIA	+	-
*lmo0448*	Similar to amino acid antiporter	IIIA	+	-
*lmo0446*	Similar to penicillin acylase and to conjugated bile acid hydrolase	IIIA	-	-
*lmo0754*	Weakly similar to a bile acid 7-alpha dehydratase	IIIA	-	-
**Transcriptional regulation**				
*lmo0041*	Similar to transcription regulator, RpiR family	IIIB	-	-
*lmo0360*	Similar to transcriptional regulator, DeoR family	IIIA	+	-
*lmo0749*	Hypothetical protein	IIIA	+	-
*lmo0753*	Similar to transcription regulator, Crp/Fnr family	IIIA	-	-
*lmo1030*	Similar to transcription regulator, BglG family	IIIA	+	-
*lmo1060*	Similar to 2-component response regulator	IIIA	+	-
*lmo2138*	Similar to transcription regulator, BglG family	IIIA	+	-
*lmo2144*	Similar to transcription regulator, GntR family	IIIA	-	-
*lmo2408*	Similar to repressor protein	IIIA	+	-
*lmo2732*	Similar to transcription regulator, RpiR family	IIIA	-	-
*lmo2773*	Similar to transcription antiterminator	IIIA	+	-
*lmo2851*	Similar to transcription regulator, AraC family	IIIA	+	-
**Transport and binding**				
*Lmo1063*	Similar to ABC transporter (ATP binding protein)	IIIA	+	-
*Lmo1100*	CadA, cadmium resistance protein	IIIA	+	-
**Translation**				
*Lmo0849*	Similar to amidases	IIIB	+	-
**Function unknown**				
*lmo0072*	Hypothetical protein	-	-	-
*lmo0086*	Hypothetical protein	IIIA	+	-
*lmo0094*	Hypothetical protein	IIIA	+	-
*lmo0095*	Hypothetical protein	IIIA	+	-
*lmo2846*	Similar to B. subtilis YulD protein	IIIA	+	516
*lmo1036*	Hypothetical protein	IIIA	-	166
*lmo0444*	Hypothetical protein	IIIA	+	-
*lmo0765*	Hypothetical protein	IIIA	+	-

^1^Genes conserved in all LI and LII genomes but absent in two or more LIII sub-groups (IIIA, IIIB or IIIC). Genes are listed based on their annotation in functional groups.

^2^LIII subgroup in which a listed gene is present.

^3^Presence "+" or absence "-" of a gene in *L. innocua *genome.

^4^Genes belong to an annotated operon based on [45]; "-", not annotated in operons.

The second-largest functional group of DDGs consists of 12 putative transcription factors representing 7 different regulatory gene families. Six are adjacent to PTS genes and possibly involved in regulating carbohydrate metabolism. Four are absent from the non-pathogenic *L. innocua *[43], *L. welshimeri *[48] and *L. seeligeri *[49], suggesting roles in virulence and pathogenicity. One Crp/Fnr (cyclic AMP receptor protein--fumarate and nitrate reduction regulator) family gene *lmo0753 *was found to be highly specific to LI and LII but absent in LIII. This Crp/Fnr factor is adjacent to a bile resistance gene *btlB *and shares similar functional domains with *prfA*, the master regulatory gene of *L. monocytogenes *virulence.

We found multiple DDGs associated with gastrointestinal (GI) tract adaptation. For instance, two bile-associated genes *btlB *(*lmo0754*) and *pva *(*lmo0446*) are absent in LIII. Both genes help *L. monocytogenes *resist the antimicrobial effects imposed by bile salts during its passage through human GI tract [50]. Loss of these genes lowered tolerance to bile and reduced persistence in murine GI tract [51]. The glutamate decarboxylase (GAD) system mediates the acid resistance in bacteria [52-54]. In *L. monocytogenes gadD1 *(*lmo0447*) is responsible for growth at mild acidic conditions (pH = 5.1) and *gadD2 *(*lmo2363*) primarily mediates the resistance to severe acidic stress (pH = 2.8) [55]. We found that *gadD2 *is conserved in all lineages, whereas *gadD1 *and its coupled glutamate: γ-aminobutyrate antiporter *gadT1 *(*lmo0448*) are absent in most LIII strains except for J2-071 and HCC23. An arginine deminase (ADI) system (*lmo0036-0041*) was recently characterized in *L. monocytogenes *[56]. The ADI system plays a role in listerial acid tolerance and may contribute to the enhanced adaptation to acidic conditions in the stomach. It was previously reported that this gene cluster is present in LI and LII but absent from LIII and non-pathogenic *L. innocua *and *L. welshimeri *[56]. Our results, however, showed that the ADI gene cluster is also highly conserved in LIIIB. An additional seventeen DDGs have no homolog in the genome of *L. innocua*, including three putative genes encoding LPXTG surface proteins (*lmo0333*, *lmo1666 *and *lmo2085*) and *sepA*, a putative virulence factor co-regulated by PrfA and σ^B ^[57,58].

### Small regulatory RNAs

Complete tiling of the *L. monocytogenes *pan-genome allowed us to survey the distribution of 100 non-coding small regulatory RNAs with specified 5' and 3' positions [45] in 9 LIII genomes. The majority (87%) of these sRNAs are conserved in LIII genomes, and only eight were found to be absent or divergent in LIII (PF < 0.6) (Table 6). Noticeably, all eight sRNAs are also absent from *L. innocua*, and five were differentially expressed in intestinal lumen or blood, suggesting roles in host niche adaptation. For example, *ril38 *contributes to listerial survival in human blood [45].

**Table 6:** Small regulatory RNAs absent or divergent in LIII genomes

RNA	**Regulation**^**1**^	**Distribution in lineage III**^**2**^								
		
		IIIA			IIIB			IIIC		
		J2-071	F2-501	F2-524	J1-208	W1-111	M1-002	F2-569	F2-208	W1-110
rli62	n/a	-	-	-	-	-	+	-	-	-
rliG	n/a	-	-	-	-	-	-	-	+	-
rli38	↑ in broth & blood	+	-	-	-	-	-	-	-	+
rli48	↑ in intestine	-	-	-	-	-	+	-	+	-
rli26	↑ in blood	+	+	+	-	-	-	-	-	-
rli29	↑ in intestine & blood	-	-	-	+	-	+	+	-	-
rli49	n/a	-	-	-	-	-	-	-	-	-
rliC	↓ in blood	+	+	+	-	-	-	-	-	+

^1^Up-regulated "↑", or down-regulated "↓" *in vivo *[45]; n/a, information not available.

^2^Gene is either present "+" or absent "-" in a LIII genome.

### Phylogenomic reconstruction

**Figure 4:**
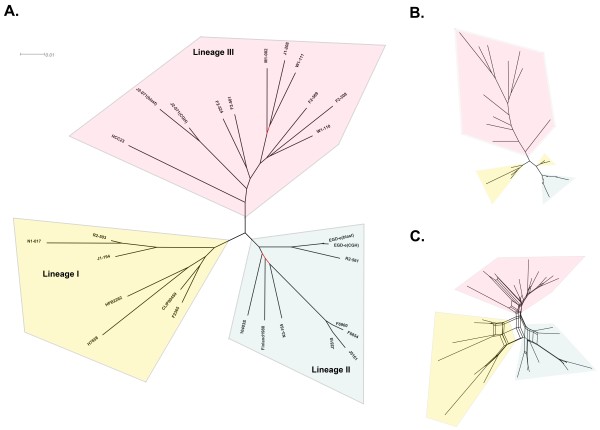
**Phylogenomic reconstruction of 26 *L. monocytogenes *strains**. (*A*) Neighbor joining (NJ) tree based on the presence or absence of 3,560 HGs in 7 LI, 9 LII and 10 LIII genomes. EGD-e and J2-071 are analyzed by both BLAST and CGH data. Braches with bootstrap (1,000 replicates) values less than 70% were labeled in red. (*B*) NJ tree based on the presence or absence of 2,855 EGD-e core genes. (*C*) Split network based on the distribution of 3,560 HGs in 26 *L. monocytogenes *genomes.

To reconstruct the phylogeny of all *L. monocytogenes *strains analyzed in this study, we surveyed the binary distributions of 3,560 HGs (Additional file 4) and 2,846 EGD-e protein-coding genes among the 26 *L. monocytogenes *genomes, respectively. We then constructed neighbor-joining (NJ) trees [59] based on a maximum-likelihood gene content distance measurement [60] (Figure 4). The NJ trees based on 3,560 HGs (Figure 4A) and 2,846 EGD-e genes (Figure 4B) both clearly separated all *L. monocytogenes *strains into 3 main clusters (i.e. a LI cluster, a LII cluster and a LIII cluster)[61]. However, the EGD-e gene-based NJ tree showed a distorted topology, indicative of a bias caused by a restricted set of loci used for phylogenetic reconstruction [62].

Of note in LI, the serotype 4b strain N1-017 appears to be closely related to serotype 1/2b strains in the LI cluster, likely representing an evolutionary intermediate between the split of serotype 4b and serotype 1/2b [10]. Of note in LII, four strains F6900, F6854, J2818 and J0161 were previously traced back to a single food processing facility over a time span of 12 years [63]. These four isolates are clustered closely on a single branch, indicative of a recent common ancestry.

While the NJ trees based on gene content allowed some inference of *L. monocytogenes *phylogeny, the reliability of the tree topology can be compromised by reticulate events such as horizontal gene transfer (HGT). Therefore, a split network was constructed using the Neighbor-net algorithm [64] to evaluate the extent by which incompatible phylogenetic signals (e.g. HGT) might affect our estimation of phylogenetic topology. Split networks do not force the formation of a tree-like structure and are able to represent incompatible signals as parallel edges, indicating the possibility of HGT or recombination. The resulting split network (Figure 4c) shows a congruent topology with the NJ tree (Figure 4a), suggesting the majority of the 3,560 HGs have been vertically inherited.

### Genomic diversification in *L. monocytogenes *lineage III

**Figure 5:**
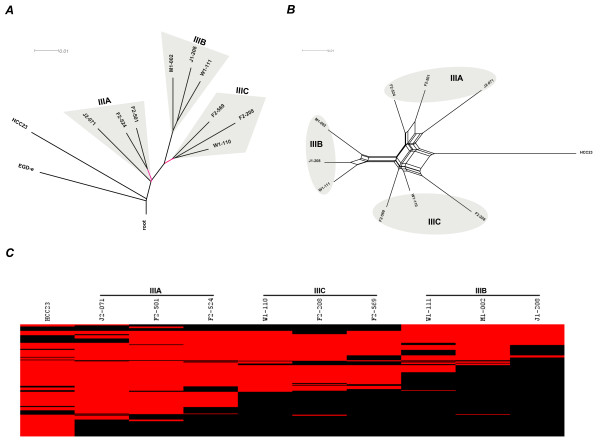
**Phylogenetic analysis of the three LIII subgroups**. (*A*) A rooted tree shows the phylogenetic relatedness of the 9 LIII strains analyzed by CGH and 1 sequenced LIII strain HCC23. The tree was rooted by EGD-e and reconstructed based on the presence or absence of 3,560 HGs using the maximum-likelihood gene content method. Two branches with bootstrap values lower than 70% (1,000 replicates) are highlighted in red. (*B*) Neighbor-net split network shows the phylogenetic relatedness of 10 LIII strains. (*C*) A heat map based on PF values shows the distribution of 206 phylogenetically informative LI and LII core genes in 10 LIII strains.

Figure 5A shows a rooted NJ tree for the three LIII subgroups, using EGD-e as an outgroup. HCC23 appears to be most closely related to LIIIA. Further evidence that links HCC23 to LIIIA is the rhamnose utilization gene cluster. This gene cluster is conserved in LIIIA and HCC23 but absent in LIIIB and LIIIC. The rooted NJ tree also suggests that LIII is polyphyletic and HCC23 possibly resembles an ancestral state of LIII. The emergence of 3 LIII subgroups is likely to be concomitant with stepwise genome reduction as observed in some non-pathogenic *Listeria *species, including *L. welshimeri *[48] and *L. seeligeri *[49].

A total of 206 genes, that are highly conserved in LI and LII, are found to be phylogenetically informative for LIII (i.e. present or absent in at least one LIII strain) (see Additional file 5). Figure 5C shows a heat map of these genes in the ten LIII strains. Interestingly, gradual gene decay or diversification was observed in the order of LIIIA, LIIIC and LIIIB. Loss of select LI and LII core genes was most significant in LIIIB. This LIII subgroup forms a deep branch in a split network (Figures 5B). However, it should be noted that the contribution of novel LIII genes to the phylogenetic reconstruction is likely to be underestimated due to the limited number of fully sequenced LIII genomes available at the time of this study.

To access the inter-lineage diversity from a gene content perspective, we identified 576, 521 and 489 accessory genes in F2365 (LI), EGD-e (LII), and J2-071 (LIII), respectively and surveyed their distributions in 26 *L. monocytogenes *genomes (Additional file 6). Minimum spanning trees were then built to compare and visualize the different distributions of these accessory genes across the three lineages (Additional file 7). Accessory genes display similar distributions in most LI and LII strains, featured by one to two dominant subsets (shown as large circles) generated by genes present or absent in most strains of the same lineage. However, more complex and branched distributions were observed in LIII strains, demonstrating an elevated genomic diversity in this rare *L. monocytogenes *lineage.

## Discussion

Pan-genome CGH was used in this study to compare *L. monocytogenes *genomes in pursuit of novel genes that potentially promote the fitness and virulence of LI and LII strains in human, as these strains are predominantly associated with human listeriosis. We used phylogenomic concepts [65] to guide our search for DDGs and to infer the phylogeny for the species. Array CGH is suitable for the purpose of this study because it is relatively cost-effective compared to the sequencing and closure required to make accurate gene calls using whole-genome shotgun sequencing. Unlike whole-genome sequencing, however, the CGH approach has several inherent limitations in detecting novel genes or pseudogenes, inferring sequence-based phylogenies, and for a host of other analyses inaccessible with array data.

A particular challenge in this study was to unify the analysis of both genome sequence and CGH array data. The sensitivity of the two methods is fundamentally different. BLAST searches are capable of precisely measuring amino acid similarity and can identify orthologs and detect distant homologies. In contrast, DNA array hybridizations measure nucleotide conservation and are only capable of detecting highly conserved DNA sequences. In addition, hybridization gives no positional information and is non-specific, making it difficult to discriminate between paralogs. For this reason, we used homologous groups for gene content comparison, and permitted variant sequences to hybridize to their nearest neighbor in a group, rather than a single selected variant (see Methods). Prior to implementing this method, there was tremendous detection bias in the CGH data. The HG method greatly increased the agreement between the array and BLAST detection strategies, which was critical for the phylogenetic analysis of the combined data.

The low frequency of LIII in human listeriosis can be partially explained by its overall rarity in foods, lack of unrecognized virulence factors, or defective mutation in some known virulence factors. For instance, a novel streptolysin S-like hemolytic and cytotoxic virulence factor, listeriolysin S, was recently found to be exclusively present in LI strains [66]. This factor contributes to virulence of the pathogen in murine and human polymorphonuclear neutrophil-based assays [66]. Several studies also reported that premature stop codons are common in *inlA *in LIII strains [67-70]. Point mutations in *inlA *are presumably caused by localized recombination and lead to a truncated InlA protein and consequently a reduced invasion phenotype in human intestinal epithelial cells [67-70]. Our pan-genome study uncovered 86 DDGs and 8 non-coding small RNAs that are absent or mutated in the largely uncharacterized LIII genomes (Table 5 and Table 6). Most of these genes fall into the functional categories of cell wall structure, transcription regulation, and carbohydrate metabolism and transport. Such functions are likely to play critical roles in ecological fitness of *L. monocytogenes *in different environment such as food processing facilities and host niches. Genes involved in carbohydrate metabolism and transport stand out as the largest functional group of DDGs, implying that the capability of utilizing different carbon sources in the transmission and infection cycle contribute most to the predominance of LI and LII strains in human infections. In particular, PTS systems that are likely to confer niche-specific metabolic advantages are conserved in LI and LII but decayed or lost in LIII. For example, the fructose-like PTS components (*lmo2133*---*lmo2137*) are conserved in all LI and LII genomes but completely lost in LIIIB and LIIIC (Figure 1). This operon was postulated to have been acquired by *L. monocytogenes *through HGT from *Enterobacteriaceae *that cohabitate the GI tract of mammalian host [71]. A recent study of its homolog in extraintestinal pathogenic *E. coli *suggested that this operon promotes bacterial fitness against the stress in host serum and gut, and enhances bacterial invasion in eukaryotic cells [72]--both are integral parts of listerial pathogenesis.

*L. monocytogenes *possess extraordinary capabilities for sustaining harsh conditions during its residency in the environment (e.g. it can utilize limited carbon source), in foods (e.g. it can resist salts and grow at refrigeration temperatures), and in parasitized hosts (e.g. it can escape from immune defense). During its passage through the human GI tract, *L. monocytogenes *is able to resist the antimicrobial effects imposed by gastric contents. Multiple genes involved in combating GI tract-related stresses, primarily gastric acid (*gadD1*, *gadT1 *and the ADI system) and bile salts (*btlB *and *pva*), are missing in LIII. Loss of these genes may result in a defective phenotype in surviving the GI tract prior to invasive infection [50]. Also absent in most LIII genomes are a number of small regulatory RNAs (e.g. *rli29 *and *rli48*) and transcription factors (e.g. *lmo2138 *and *lmo2851*) that appear to be up-regulated in the murine intestine [45]. It is reasonable to speculate that the GI tract may act as a major barrier to prevent LIII strains from causing systematic infections. Epidemiological studies seem to support this speculation by collectively showing that gastroenteritis, rather than more severe listeriosis symptoms, is predominant among infected individuals [73-75]. Although intracellular strategies have been the primary focus in numerous studies of listerial pathogenesis, a few recent studies demonstrated that the GI passage has a fundamental impact on listerial pathogenicity [76,77]. Considering that most LIII strains possess virulence factors related to its intracellular lifestyle and are cytopathogenic [14], the inability to survive in the GI tract becomes a plausible explanation for the overall rarity of LIII in human listeriosis.

We estimate that the *L. monocytogenes *core-genome consists of 2,330 to 2,456 genes and the pan-genome encompasses over 4,052 genes (Figure 3). Compared to several other bacterial species, *L. monocytogenes *has relatively higher proportions (about 80%) of core genes shared by individual genomes (Table 7), which in turn reflects lower intraspecies genomic variability. This is consistent with the low rates of recombination in this bacterial species [68]. Despite the perceived high genomic synteny, *L. monocytogenes *possesses considerably diverse pan gene reservoir and displays biased distribution of accessory genes across major evolutionary lineages (Additional file 7).

**Table 7:** Summary of pan-genomic studies

Species	**No. Genomes**^**1**^	**Pan genome**^**2**^	No. core genes	No. pan genes	Avg. no. genes	% Core genes	**Blast cutoff**^**3**^	Ref
*Escherichia coli *&*Shigella*	20	Open	1976	> 17838	4700	42%	80/80	[33]
*Escherichia coli*	17	Open	2200	> 13000	5020	44%	0.8 BSR	[32]
*Escherichia coli*	32	Open	1563	> 9433	4537	34%	50/50	[31]
*Haemophilus influenzae*	13	Finite	1461	4425-6052	1970	74%	70/70	[25]
*Listeria monocytogenes*	26	Open	2350-2450	> 4000	2978	80%	0.5 SSR	This study
*Neisseria meningitides*	7	Open	1333	> 3290	1963	68%	50/50	[30]
*Streptococcus agalactiae*	8	Open	1806	> 2750	2245	80%	50/50	[24]
*Streptococcus agalactiae*	8	*Open	1472	*> 2800	2198	67%	1e-5 E-value	[29]
*Streptococcus pneumoniae*	17	Finite	1380	5100	2438	57%	70/70	[28]
*Streptococcus pyrogenes*	11	*Closed	1376	*2500	1878	73%	1e-5 E-value	[29]

All numbers are estimates in this table.

^1^Only studies including more than five strains are shown.

^2^Pan-genome growth behaviors as described by the authors. * Estimated from figures, but not explicitly stated in the paper.

^3^Cutoff values and methods for defining core and pan genes vary widely across the different studies. This column only gives a rough summary of the similarity cutoff. Cutoffs of the form *I*/*L *indicate a minimum BLAST hit of *I*% similarity over *L*% of the protein length. BSR is Blast Score Ratio [32]. SSR is the similarity score ratio used in this study, similar to BSR.

Some incompatible phylogenetic signals as indicated in the split network (Figure 4C) were traced back to prophage-associated genes. Notably, the *comK *prophage regions in different *L. monocytogenes *genomes display significant sequence variations (Additional file 8). Such variations may be a result of prophage decay, recombination that have accumulated in the remnants of common prophage ancestor(s), or multiple lysogenization of different bacteriophages at the same genomic location. Phages have been recognized as the major contributors of important biological properties (e.g. virulence factors) in many bacterial species [78,79]. The functional impact of bacteriophages on the biology of *L. monocytogenes*, if any, has yet to be determined.

## Conclusions

Intraspecific variations in host preference, ecological fitness and virulence are common in many bacterial pathogens. This is exemplified by the species of *L. monocytogenes *which consists of multiple distinct genetic lineages. Two lineages of this species (i.e. LI and LII) predominantly cause human sporadic and epidemic infections, whereas the other (i.e. LIII) has never been implicated in human disease outbreaks for unclear yet intriguing reasons. Here we described a novel pan-genomic approach that combines *in silico *comparative analysis and high-density CGH arrays to explore the genomic diversity of *L. monocytogenes*. Our integrated approach allows vigorous core genome estimation and phylogenomic reconstruction, which in turn is nearly impossible for low-quality, short-read draft genome assemblies with hundreds of contigs. Exponential regression analysis predicts that *L. monocytogenes *has a core genome of between 2,330 to 2,456 genes (80% of each individual genome) and a pan-genome repertoire of over 4,052 unique genes. Comparison of all lineage strains reveals high genomic synteny with limited sequence drift associated with lysogenic bacteriophages. Phylogenomic reconstructions based on 3,560 homologous groups suggest a polyphyletic population infrastructure and gradual loss of metabolic genes as this saprophytic species diversified into the rare and probably defective lineage III. Based on our results, one *L. monocytogenes *strain carries about 75% of the pan genes of this species. That said, experiments based on a single reference strain may not adequately sample the total genetic repertoire and not fully interpret the versatile biology of *L. monocytogenes*. With a more defined species core genome, we may also be able to supplement new genomic criterion for taxonomic classification of *L. monocytogenes*, as some traditional methods are often inconclusive and controversial. The pan-genomic approach described here can be used to explore the genomic diversity in other pathogenic species, as such information would be extremely valuable for us to better understand the intraspecific variations in virulence, and the ecology, epidemiology and evolution of microbial pathogens.

## Methods

### Bacterial isolates and genomic DNA extraction

Table 1 lists the 31 *L. monocytogenes *strains analyzed in this study. As of November 2008, twenty sequenced *L. monocytogenes *strains were available and used for the pan-genome array design. CGH was performed for nine LIII strains representing 3 serotypes (4a, 4b, and 4c) and 3 subgroups (LIIIA, LIIIB, and LIIIC). Four additional isolates that were sequenced after the array design were incorporated in the pan-genomic and phylogenetic analysis. Bacterial strains were grown overnight in brain heart infusion (BHI) broth at 35°C. Genomic DNA was extracted and purified using MasterPure Gram positive DNA purification kit (EPICENTRE Biotechnologies, Madison, WI). Genomic DNA was labeled with Cy3 or Cy5 dye prior to array hybridization.

### Pan-genomic array design

The pan-genome tiling array was designed using the PanArray software [38] to fully tile the 20 sequenced *L. monocytogenes *genomes (Table 1). PanArray employs a greedy probe selection algorithm to tile multiple whole genomes using a minimal number of probes. For this study, PanArray was used to design an array comprising 385,000 50-mer oligonucleotide probes that fully tile the 20 listerial genomes at 2.65 × coverage with no gaps. To avoid tiling low quality or contaminant sequence, contigs less than 2 Kbp in length were discarded, leaving 54,810,759 bp of tiled sequence. A full description of the array design is given in [38], and the array design is available from the Gene Expression Omnibus (GEO) [80] under accession number GPL8942. To incorporate newly sequenced strains that had not been included in the original array design, we aligned all probes on the array to the new genomes allowing one mismatch per probe, and added genes with probe coverage ≥ 90% of their length to the array annotation.

### Array hybridization and data analysis

Genomic DNA of each LIII strain was co-hybridized with that of EGD-e on a Roche NimbleGen 385 K custom CGH array. Two dye-swap replicates were performed for each LIII strain/EGD-e pair to eliminate dye bias and test the array reproducibility. Genomic DNA labeling and array hybridization were performed at Roche NimbleGen according to the manufacturers specifications (Madison, WI). Technical details on DNA labeling and hybridization can be found at http://www.nimblegen.com/products/lit/cgh_userguide_v6p0.pdf . We designed a probe-based intensity classification scheme to provide the most flexibility for pan-genome array data analysis, allowing any locus to be classified based on the aggregated scores of its individual probes, without reference to control hybridization. Specifically, all raw signal intensities were first transformed to log values, then log intensities for replicate hybridizations were normalized using quantile normalization [81]. Replicates were combined at the probe level by taking the average of the normalized log intensities for each probe. Quantile normalization assumes similar intensity distributions, so to avoid cross-sample normalization bias. Each strain was normalized and processed independently.

Because there was no one single reference to operate on, and to preserve sensitivity for small polymorphisms, intensity data was not smoothed or segmented. Instead, individual probes were each classified as present or absent using a minimum kernel density (MKD) method. MKD methods have performed well for the binary classification of both genes and segments [31,82], and here we extended the idea to the classification of individual probes. Because the array contains the full genetic diversity of *L. monocytogenes *and 4,300 random control probes, there is expected to be a significant fraction of both present and absent probe intensities for any *L. monocytogenes *sample. Therefore, the distribution of probe intensities is generally bimodal, and the minima between the present and absent peaks can be used as an effective threshold for binary classification. For each sample, the probability density function of the observed intensities was estimated using kernel density estimation and the central minima of this function identified as the optimal cutoff (Additional file 9). This method was preferred because it is non-parametric, there is no potential normalization bias, it requires no training, and each sample can be processed independently without affecting the accuracy. It is also extremely flexible, in that a classification for any gene can be generated by aggregating the classifications of the probes targeting that gene. For this purpose, genes were scored by collecting all probes known to target a specific gene and computing the fraction of probes classified as present, the positive fraction (PF). A PF threshold of 0.6 was chosen by analysis of ROC curves for the EGD-e and J2-071 controls to minimize the total error rate (false-positive rate + false-negative rate) versus the tblastn 50% protein similarity threshold. PF was favored because it does not depend on cross-sample normalization, as would be necessary for an intensity threshold, and additional genomes can be analyzed independently without affecting accuracy. This makes it ideal for rapid and economical analysis of novel isolates, while maintaining comparable accuracy to alternative analysis methods [31,34].

### Pan-genomic analysis

Pan-genomic analysis was performed using the methods introduced by Tettelin *et al. *[24], with modifications on the conservation threshold and permutation sampling. Annotated proteins for each genome were aligned to the six frame translations of all other genomes using tblastn. Query proteins were marked as present in a subject genome if the corresponding amino acid sequences aligned at ≥ 50% similarity with an *E*-value ≤ 10^-5^, where "similarity" was defined as the number of positively scored residues divided by the length of the protein sequence. This threshold is more stringent than originally proposed in [24], but less stringent than those used in other studies (e.g. [32]). The 50% threshold was empirically selected as a compromise between tolerating draft genomes with fragmented annotations and avoiding false positive detections due to conserved domains and distant paralogs. A PF threshold of 0.6 was consequently chosen as an analogous threshold for the CGH results, as described above. Genomes sequenced to less than 10× coverage using 454 pyrosequencing were excluded from the analysis.

The addition of an *N*^th ^genome was simulated by examining ordered combinations of *N *genomes. Due to the large number of available genomes, it was not feasible to consider all possible permutations as originally suggested. Instead, a randomly selected subset of 100,000 permutations was considered for the addition of each *N*, and the mean (or median) values were computed from this subset. For each permutation, the number of new genes found in the *N*^th ^genome *G*_*N *_was computed as the number of proteins of *G*_*N *_not present in any genomes *G*_*i *_for *i *= {1, ..., *N*-1}. The number of core genes was computed as the number of proteins of *G*_*N *_present in all genomes *G*_*i *_for *i *= {1, ..., *N*}. Because gene sequences for the CGH strains are not known, EGD-e was set to be *G*_*N *_for all permutations. The number of pan genes in a permutation of *N *genomes was computed by examining the genomes *G*_*i *_in order from 1 to *N*. A gene in *G*_*i *_was identified as a pan gene if it was not present in any of the genomes *G*_*j *_for all *j < i*.

The Gauss-Newton method implemented by the R function *nls *[83] was used to perform non-linear least squares regression on the mean and medians of the core genes, new genes, and pan genes distributions. According to [26], the number of new genes *n *expected to be discovered by sequencing an *N*^th ^genome was modeled by the power law function *n *= *κN*^-*α*^, and the number of pan genes also by a power law *n *= *κN*^*γ*^. According to [24], the number of core genes was modeled by the exponential decay function *n *= *κe*^-*N*/*τ *^+ Ω, where Ω describes the horizontal asymptote and therefore the core genes estimate. In all cases, the functions were fit to the mean or median values for all *N *> 1.

To accommodate false-negative errors introduced by sequencing gaps and weak hybridization signal, the originally proposed exponential decay function was modified with the addition of a fourth parameter to model the effect of a constant number of false-negatives with the addition of each genome. The modified equation is:

$$n=\kappa e^{-N/\tau }+\Omega -N\beta$$

where the linear parameter *β *represents the number of core genes lost to false-negative errors for each *N*. Core gene loss due to false-negatives is not a truly linear phenomenon (e.g. sequencing gaps are not independent and the core genome can never be negative), but for a large core genome and a modest *N *it is a reasonable approximation that is easy to fit. To assure convergence of the optimization algorithm, *β *was first estimated via linear regression for *N *≥ 15, and this was used as the start estimate of *β *for the full model regression. The augmented model is useful in that the observed core genome size may be linearly decreasing (as is expected for draft genomes), but an estimate of the true core genome size Ω may still be recovered.

### Identification of homologous groups

Homologous groups (HGs) were used for phylogenetic reconstruction and core genome estimation. HGs were identified by clustering a graph of protein similarity for all annotated protein-coding genes from the 18 high-quality *L. monocytogenes *genomes. A node was added to the graph for each one of the 52,776 annotated proteins. Edges were added between any two proteins with an alignment above the 50% similarity threshold. Unlike OrthoMCL, no orthology constraint was applied. Edges between any two similar proteins were added, including edges between proteins in the same genome. This was necessary due to the inability of CGH to accurately determine orthology. The MCL clustering algorithm was applied to this graph using an inflation parameter of 2.0. From this clustering, 3,744 HGs were identified, including strain-specific genes represented as singleton clusters (Additional file 2). Some HGs, mostly singletons, were not represented on the array because additional genomes had been sequenced after the array design. A total of 3,560 HGs, represented on the array by at least one member gene, were used for the phylogenetic analysis.

For sequenced genomes, an HG was called present if at least one member protein of the HG aligned above the 50% similarity threshold. For CGH genomes, an HG was called present if at least one member gene of the HG hybridized with PF ≥ 0.6. Results based on this threshold were converted to a unified binary table indicating gene presence or absence for all HGs in all genomes analyzed in this study (Additional file 4). These binary vectors were used for measuring evolutionary distance using the maximum-likelihood measure of [59], and Neighbor-net split networks [60] and neighbor-joining trees [65] were built using the SplitsTree program [84]. Alternative parsimony methods failed to build reasonable trees, most likely due to the large number of incompatible splits caused by both horizontal gene transfer and errors in the data.

### PCR verification of lineage-specific genes

PCR primers were designed for 3 LI and 6 LIII specific genes using the Primer3 software (available at http://frodo.wi.mit.edu/primer3/). Colony PCR for each gene was performed for 8 LI, 8 LII and 9 LIII strains using the Taq Mastermix PCR kit (Qiagen, Valencia, CA). PCR amplicons were confirmed by the proper size of the DNA bands after agarose gel electrophoresis.

### Data accession

Hybridization results have been deposited at the NCBI Gene Expression Omnibus under accession number GSE20367.

## Abbreviations

LI: genetic lineage I; LII: genetic lineage II; LIII: genetic lineage III; LIIIA: genetic lineage III subgroup A; LIIIB: genetic lineage III subgroup B; LIIIC: genetic lineage III subgroup C; CGH: comparative genomic hybridization; HGT: horizontal gene transfer; HG: homologous group; DDG: disparately distributed genes; PTS: phosphotransferase system; GI: gastrointestinal; GAD: glutamate decarboxylase; ADI: arginine deminase system; MKD: minimum kernel density; PF: positive fraction; ROC: receiver operating characteristics.

## Authors' contributions

Conceived and designed the experiments: XD WZ. Designed the array and analysis methods: AMP. Performed the experiments: XD. Analyzed data: XD AMP ZL. Coordinated the project: SLS WZ. Wrote the paper: XD AMP WZ. All authors read and approved the final manuscript.

## Supplementary Material

Additional file 1 **Density estimation of PF values for both present and absent genes**. Barplot of the positive fraction probability densities for known present and absent genes demonstrates the vast majority of truly present genes have PF score greater than 0.9 and the vast majority of truly absent genes have PF less than 0.1. Green bars show the density of PF scores for genes found present by a tblastn search, and black bars show the density of PF scores for genes found absent by a tblastn search. PF labels give the minimum of each left-closed interval. For example, PF = 0.5 bars show the densities for the bucket PF = [0.5,0.6). Click here for file

Additional file 2 **Homologous groups with locus tax IDs in 26 *L. monocytogenes *genomes**. A total of 3,744 homologous groups were identified from 52,776 proteins annotated in 26 *L. monocytogenes *genomes. Each row in this file indicates the locus and tax IDs of proteins belonging to a specific homologous group. Click here for file

Additional file 3 **Comparison of homologous genes in rhamnose metabolic pathways**. Alignment of putative rhamnose utilization pathway in *E. coli *strain K-12, *L. monocytogenes *strain EGD-e, and *B. subtilius *strain 168. The percentage of amino acid sequence similarities is shown between homologous gene pairs. Genes encoding L-rhamnose isomerase, L-rhamnulose kinase, and rhamnulose-1-phosphate aldolase are located in the same orientation in *L. monocytogenes *EGD-e and *E. coli *K-12 genomes. The pathway is adopted from KEGG database. Click here for file

Additional file 4 **Presence or absence of 3,560 HGs in 26 *L. monocytogenes *genomes**. Binary distribution (i.e. present: "1"; absent or divergent: "0") of 3,560 HGs in 26 *L. monocytogenes *genomes. Each HG is designated by the tax and locus IDs of a representative protein. Click here for file

Additional file 5 **Distribution of phylogenetically-informative LI and LII core genes in LIII**. Binary distribution (i.e. present: "1"; absent or divergent: "0") of phylogenetically-informative LI and LII core genes in LIII strains were summarized in this table. Click here for file

Additional file 6 **Binary distribution of accessory genes in 26 *L. monocytogenes *genomes**. Binary distribution (i.e. present: "1"; absent or divergent: "0") of accessory genes in 26 *L. monocytogenes *genomes. These genes were used to generate the minimal spanning trees. Click here for file

Additional file 7 **Minimum spanning trees that show the distribution of accessory genes in different *L. monocytogenes *lineages**. A total of 576, 521 and 489 accessory genes were identified from F2365 (LI), EGD-e (LII), and J2-071 (LIII), respectively. The binary distribution of these accessory genes was surveyed in 28 *L. monocytogenes *genomes, including 4 newly sequenced strains. Each circle represents a group of accessory genes in F2365 (*A*, *D*, *G*), EGD-e (*B*, *E*, *H*), or J2-071 (*C*, *F*, *I*) that share a unique binary distribution (i.e. "1" for presence or "0" for absence) in all strains belonging to a specific lineage (i.e. I, II, or III). The size of each circle is proportional to the total number of genes that share the same binary distribution. Each circle is color-coded based on the number of *L. monocytogenes *strains (from 0 to 10, see color bar) that share the same distribution. This figure provides an overview of the genomic diversity of the three genetic lineages from a perspective of accessory gene presence or absence, where LIII displays the most diversified gene content. Click here for file

Additional file 8 **Alignment of A118-like prophage in different *L. monocytogenes *lineages**. The x-axis gives the location on the EGD-e chromosome, and for each strain, windowed alignment identity is given on a scale of 50-100% identity on the y-axis. Strains which show no homology to the EGD-e A118-like prophage are struck through in blue line. Strains which do show homology to the prophage, but the prophage is inserted somewhere other than *comK*, are struck through in red line (N1-017, HCC23). This plot illustrates some interesting phylogenetic incompatibilities. For example, based on whole-genome analysis, the nearest phylogenetic neighbor to EGD-e is R2-561. Yet the *comK *prophage in nearly all other strains appears more similar to EGD-e than does the prophage in R2-561, which has identity < 50% for most of its length. Click here for file

Additional file 9 **Probe density versus mean log intensity of the CGH arrays**. Histogram with overlaid kernel density estimation (red) of the distribution of probe intensities for sample J1-208, showing an optimal intensity cutoff of 8.82 at the minimum between the present and absent modes. Displayed distribution is for the mean intensities of the two normalized quantile replicates for strain J1-208. Click here for file
