# Supplementary figures and images for: Probing the pan-genome of Listeria monocytogenes: new insights into intraspecific niche expansion and genomic diversification
Source: BMC Genomics. 2010 Sep 16;11:500. doi: 10.1186/1471-2164-11-500 (PMC2996996; doi:10.1186/1471-2164-11-500)

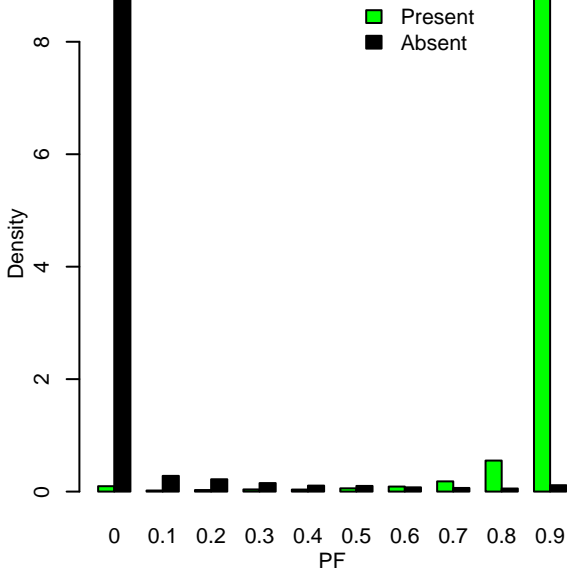

Supplement: Additional file 1 — Density estimation of PF values for both present and absent genes. Barplot of the positive fraction probability densities for known present and absent genes demonstrates the vast majority of truly present genes have PF score greater than 0.9 and the vast majority of truly absent genes have PF less than 0.1. Green bars show the density of PF scores for genes found present by a tblastn search, and black bars show the density of PF scores for genes found absent by a tblastn search. PF labels give the minimum of each left-closed interval. For example, PF = 0.5 bars show the densities for the bucket PF = [0.5,0.6). [file 1471-2164-11-500-S1.PDF]

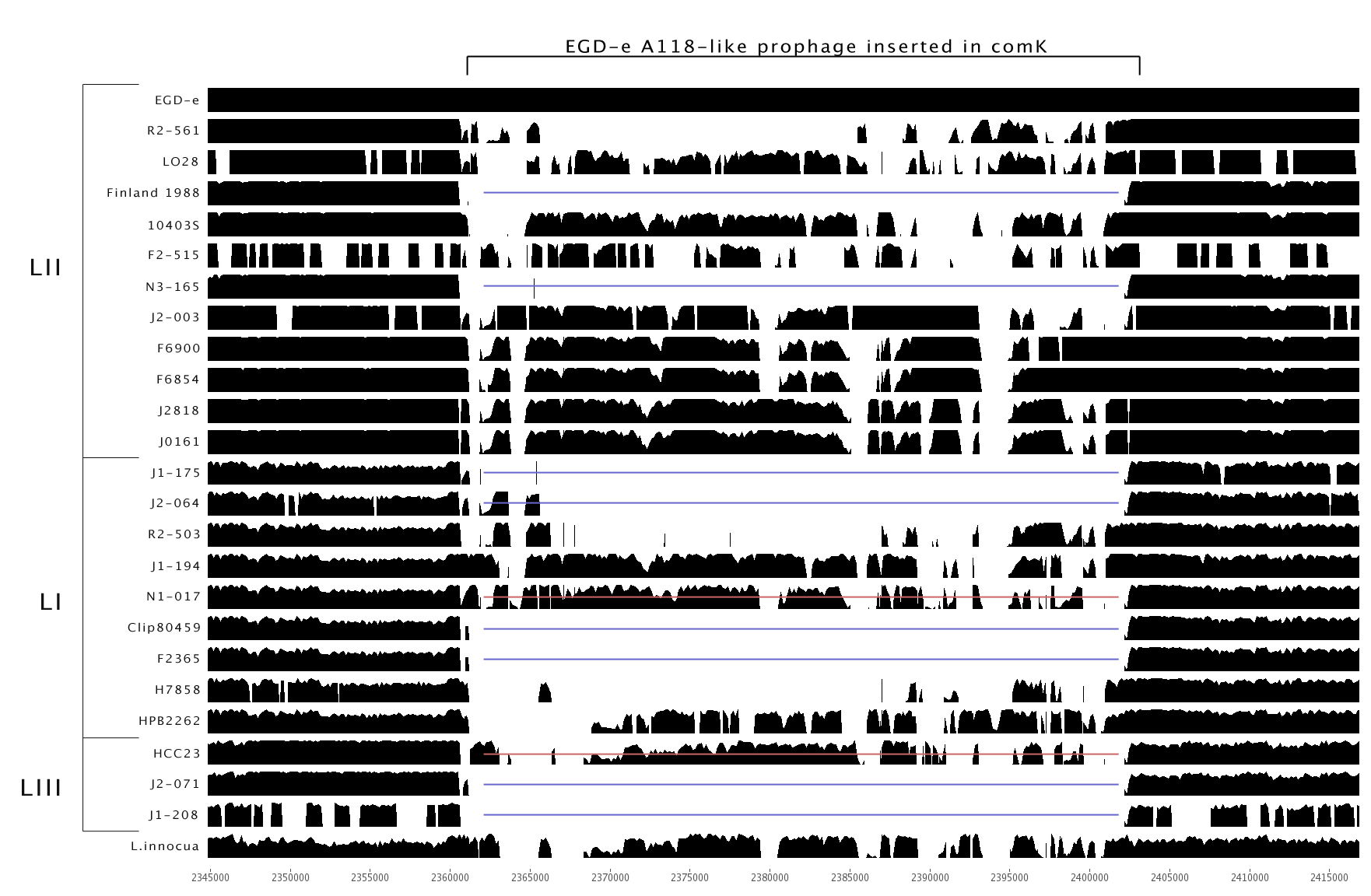

Supplement: Additional file 8 — Alignment of A118-like prophage in different L. monocytogenes lineages. The x-axis gives the location on the EGD-e chromosome, and for each strain, windowed alignment identity is given on a scale of 50-100% identity on the y-axis. Strains which show no homology to the EGD-e A118-like prophage are struck through in blue line. Strains which do show homology to the prophage, but the prophage is inserted somewhere other than comK, are struck through in red line (N1-017, HCC23). This plot illustrates some interesting phylogenetic incompatibilities. For example, based on whole-genome analysis, the nearest phylogenetic neighbor to EGD-e is R2-561. Yet the comK prophage in nearly all other strains appears more similar to EGD-e than does the prophage in R2-561, which has identity < 50% for most of its length. [file 1471-2164-11-500-S8.TIFF]

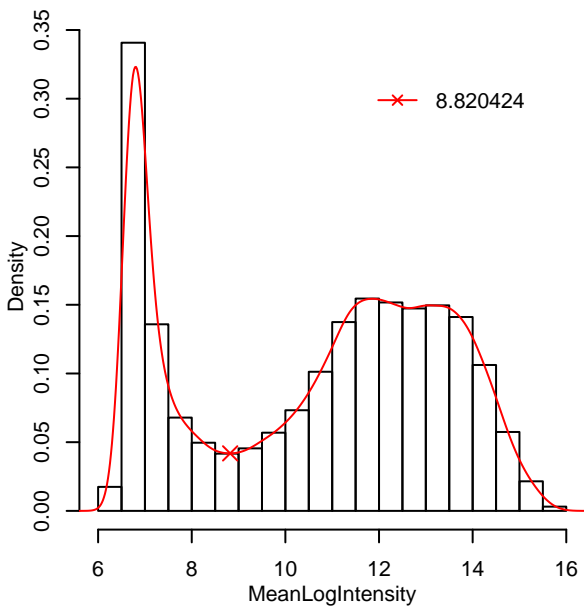

Supplement: Additional file 9 — Probe density versus mean log intensity of the CGH arrays. Histogram with overlaid kernel density estimation (red) of the distribution of probe intensities for sample J1-208, showing an optimal intensity cutoff of 8.82 at the minimum between the present and absent modes. Displayed distribution is for the mean intensities of the two normalized quantile replicates for strain J1-208. [file 1471-2164-11-500-S9.PDF]
