# Supplementary material for: Probing the pan-genome of Listeria monocytogenes: new insights into intraspecific niche expansion and genomic diversification
Source: BMC Genomics. 2010 Sep 16;11:500. doi: 10.1186/1471-2164-11-500 (PMC2996996; doi:10.1186/1471-2164-11-500)
Supplement: Additional file 3 — Comparison of homologous genes in rhamnose metabolic pathways. Alignment of putative rhamnose utilization pathway in E. coli strain K-12, L. monocytogenes strain EGD-e, and B. subtilius strain 168. The percentage of amino acid sequence similarities is shown between homologous gene pairs. Genes encoding L-rhamnose isomerase, L-rhamnulose kinase, and rhamnulose-1-phosphate aldolase are located in the same orientation in L. monocytogenes EGD-e and E. coli K-12 genomes. The pathway is adopted from KEGG database. [file 1471-2164-11-500-S3.PDF]

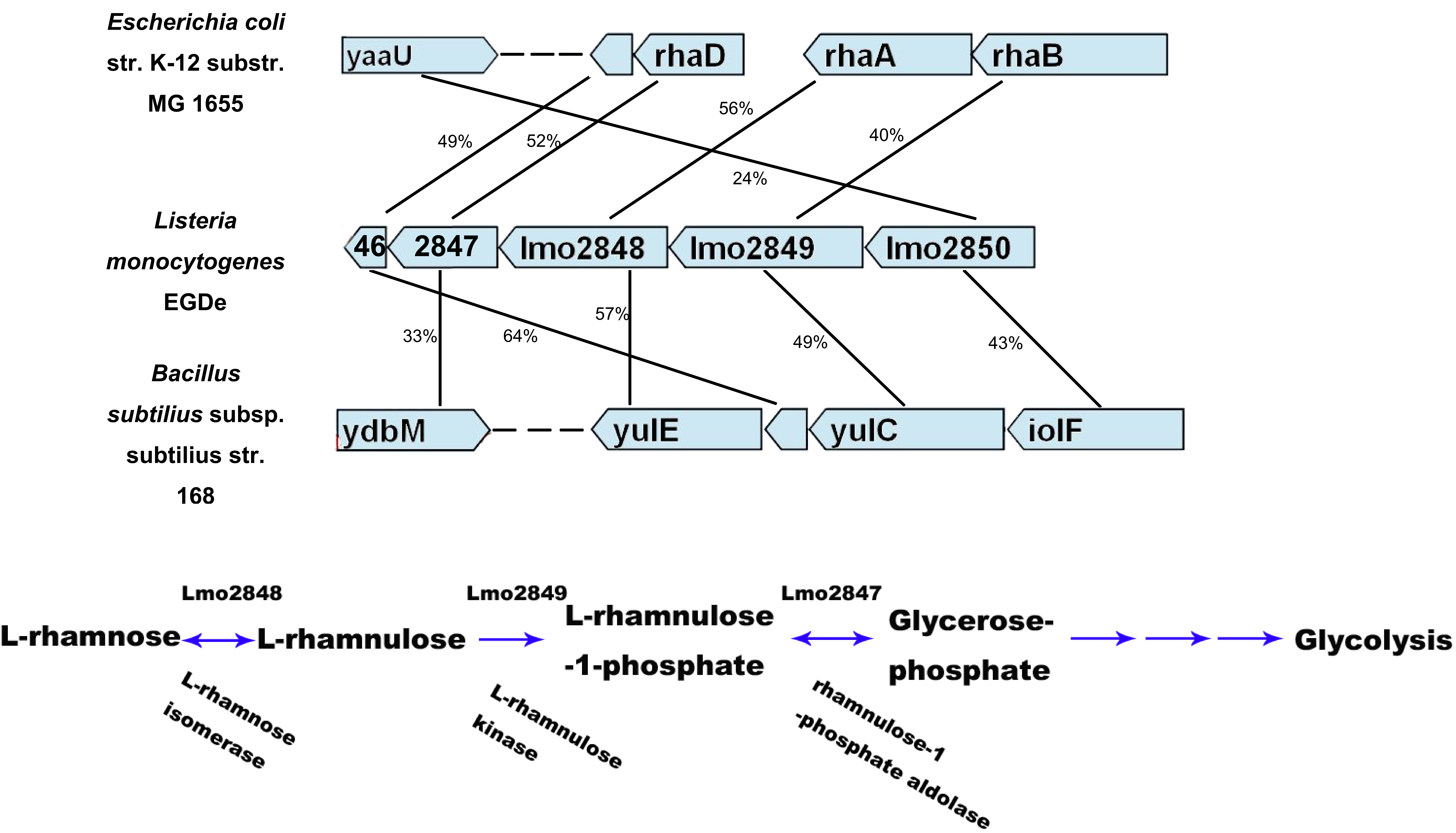

Escherichia coli str. K-12 substr. MG 1655
yaaU
rhaD
rhaA
rhaB
49%
52%
56%
40%
24%
Listeria monocytogenes EGDe
46
2847
lmo2848
lmo2849
lmo2850
33%
64%
57%
49%
43%
Bacillus subtilius subsp. subtilius str. 168
ydbM
yulE
yulC
iolF
Lmo2848
Lmo2849
Lmo2847
L-rhamnose
L-rhamnulose
L-rhamnulose -1-phosphate
Glycerose-phosphate
Glycolysis
L-rhamnose isomerase
L-rhamnulose kinase
rhamnulose-1 -phosphate aldolase
